# Prognostication After Dialysis Withdrawal

**DOI:** 10.1016/j.ekir.2024.04.045

**Published:** 2024-04-24

**Authors:** Sarah So, Kelly Chen Lei Li

**Affiliations:** 1 Department of Renal Medicine, Nepean Kidney Research Center, Nepean Hospital, Kingswood, Sydney, New South Wales, Australia; 2 Department of Renal Medicine, St George Hospital, Kogarah, Sydney, New South Wales, Australia; 3 The University of Sydney, Sydney, New South Wales, Australia

**Keywords:** chronic kidney disease, dialysis withdrawal, end stage kidney disease, kidney supportive care, renal supportive care

## Abstract

**Introduction:**

Dialysis withdrawal represents an increasingly common cause of death in patients receiving kidney replacement therapy internationally. Prognostic information about stopping dialysis guides clinicians counseling patients and families regarding end-of-life care. However, few studies examine prognostication after withdrawal. We aimed to determine median survival time after withdrawal of dialysis, and to determine which patient and dialysis-related factors are significantly associated with prognosis.

**Methods:**

This retrospective cohort study used registry data. We included all adult patients from the Western Renal Services who were receiving peritoneal dialysis (PD) or hemodialysis prior to death, whose cause of death was documented as “withdrawal from dialysis” and whose date of death was between January 1, 2016 and June 30, 2022. Demographic, clinical, and biochemical data was extracted. The primary outcome was time-to-death, defined as days from last dialysis session to date of death.

**Results:**

Median survival time from last dialysis to death for the PD group (*n* = 53) was 4 days (interquartile range [IQR]: 3–10 days), not significantly different from the hemodialysis group which was 6 days (IQR: 2–11 days, *P* = 0.72). For PD, the only variable significantly associated with survival time was reason for withdrawing (*P* = 0.01). Median survival time was significantly longer for patients withdrawing for psychosocial reasons compared to those withdrawing for other reasons (*P* = 0.002). For hemodialysis (*n* = 186), variables significantly associated with survival time from last dialysis to death was reason for withdrawing (*P* = 0.001), urine production at the time of withdrawal (*P* = 0.005), serum sodium (*P* = 0.02) and smoking status (*P* = 0.009).

**Conclusion:**

Median survival time was longer for withdrawals for psychosocial reasons compared to medical reasons. The data presented could inform withdrawal discussions regarding prognostication and end-of-life planning with patients and family.

## Graphical abstract

**Figure:**
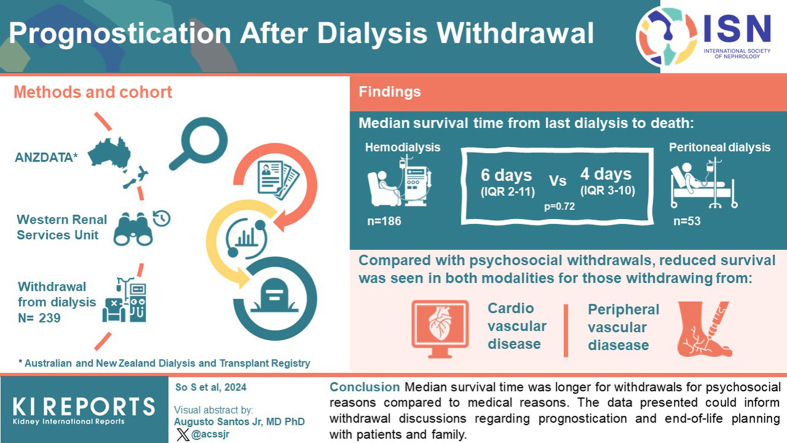

See Commentary on Page 1963

In Australia and New Zealand, dialysis withdrawal represents up to 24% of deaths in patients with end-stage kidney disease.^1^ Internationally, dialysis withdrawal is also an increasingly common cause of death in patients on kidney replacement therapy.^2^, ^3^, ^4^ Although reasons for withdrawal vary from medical to psychosocial,^5^^,^^6^ all necessitate end-of-life care planning. Prognostication in palliative care can facilitate patient-centered care, shared decision making and help patients and families plan for end-of-life care.^7^^,^^8^ Counseling about what to expect in terms of prognosis after withdrawal from dialysis is recommended in renal populations.^9^ However, few studies examine prognostication after dialysis withdrawal, and the factors affecting length of survival after withdrawal of treatment. Available studies report that median survival approximates 4 to 7 days^10^^,^^11^ after stopping hemodialysis. Factors associated with poorer survival included male sex, lower performance status, and peripheral edema^11^; and factors associated with better survival included better oral nutrition and ventilator use.^10^ However, these studies were limited by lack of renal-specific clinical data such as dialysis vintage and urine output and consisted of only patients on hemodialysis.

Existing prognostication tools used in palliative care, such as the Palliative Performance Scale or Palliative Prognostic Score, are primarily aimed at patients with malignant disease and incorporate factors that may not be relevant to patients withdrawing from dialysis.^8^ As an example, the Palliative Performance Scale assesses domains of ambulation, activity, self care, oral intake, and level of consciousness; and has not been shown to correlate with median survival in patients without cancer.^8^^,^^12^ Prognostic factors such as serum albumin, white cell count, lymphocyte percentage and comorbidities are used in these scoring tools. Furthermore, they do not include variables that are relevant to patients on long term dialysis, such as dialysis vintage or residual urine output. Conversely, existing prognostication tools for renal patients focus on their survival while they remain on hemodialysis, such as the REIN (Renal Epidemiology and Information Network) score.^13^^,^^14^ There is no similar data to help guide clinicians caring for patients who choose to withdraw from dialysis. This study aims to determine what patient and dialysis-related factors are significantly associated with prognosis after dialysis withdrawal and provide data on survival time after withdrawal of PD and hemodialysis. This will help guide clinicians when counseling patients and families electing for dialysis withdrawal in their expectations about anticipated end-of-life care.

## Methods

This retrospective cohort study is based on data that the Western Renal Services Renal Unit has systemically collected for purposes of mandatory Australian and New Zealand Dialysis and Transplant Registry submission.

### Study Population

The Australian and New Zealand Dialysis and Transplant Registry is a binational registry encompassing deidentified data for Australian and New Zealand patients receiving dialysis or kidney transplantation. Information is collected by medical and nursing staff and submitted annually to the Australian and New Zealand Dialysis and Transplant Registry. The data is deidentified in the Registry; however, each unit is able to access its own reidentifiable data. This study received approval from the Nepean Blue Mountains Local Health District Human Research Ethics Committee.

We included all adult patients (aged 18 years and over) from the Western Renal Service who were receiving PD or hemodialysis prior to death, whose cause of death was documented as “withdrawal from dialysis” and whose date of death was between January 1, 2016 and June 30, 2022. We excluded patients if they did not have a date of death recorded.

### Data Collected

Data that was collected from our unit’s Australian and New Zealand Dialysis and Transplant Registry submissions included demographic data (medical record number, sex, racial origin, date of birth, age, primary renal disease, whether they commenced dialysis less than 3 months of first being referred to a nephrologist, postcode at entry, and country of birth), dialysis data (date of commencement of dialysis, dialysis modality at commencement, and last dialysis modality recorded), height and weight at dialysis commencement, death data (date of death and cause of death) and comorbidities (cerebrovascular disease, cardiovascular disease, peripheral vascular disease, chronic lung disease, and diabetes mellitus at the last date recorded by the registry), and smoking status (former, current, or never smoked) at commencement of dialysis.

Patients were then matched to our service’s electronic medical records and further clinical and biochemical data extracted. Clinical data included last recorded weight (in kg, defined as the last weight recorded within 6 months of the patient’s death), whether the patient was still producing urine daily or not at time of dialysis withdrawal, blood pressure at time of dialysis withdrawal (defined as the last blood pressure recorded within the 24 hours preceding dialysis cessation), last date of dialysis, reason for withdrawal from dialysis, whether palliative care services were consulted within the 7 days prior to death) and Charlson comorbidity index data (myocardial infarction, congestive cardiac failure, peripheral vascular disease, cerebrovascular disease, dementia, chronic obstructive pulmonary disease, peptic ulcer disease, end-organ disease from diabetes mellitus, hemiplegia, leukemia, lymphoma, malignancy [metastatic or nonmetastatic], chronic liver disease, and ischemic heart disease). The last biochemical results were recorded from results which were collected within the last 30 days of the patients’ last dialysis session. Biochemical results included hemoglobin, white cell count, lymphocyte count, serum sodium, serum potassium, serum bicarbonate, corrected calcium, phosphate, albumin, C-reactive protein, and urea and creatinine for patients on PD.

### Statistical Analyses

The primary outcome was time-to-death, defined as days from a patient’s last dialysis session to date of death. Analyses were performed separately for the hemodialysis and the PD groups. Potential variables analyzed included demographic data, comorbidities (myocardial infarction, congestive heart failure, peripheral vascular disease, cerebrovascular disease, dementia, chronic obstructive pulmonary disease, connective tissue disease, peptic ulcer disease, diabetes mellitus, hemiplegia, leukemia, malignant lymphoma, solid tumor, liver disease, and AIDS), urine output at time of death, and biochemical data (excluding urea and creatinine for patients on hemodialysis).

Descriptive data were reported separately based on groups (PD or hemodialysis). Continuous variables were summarized with means ± SD for normally distributed variables, and medians (IQR) for non-normally distributed variables. Unpaired *t* tests were performed to compare differences in normally-distributed continuous variables between the 2 groups. Mann-Whitney U tests were performed to compare differences in non-normally distributed continuous variables between the 2 groups. Categorical variables were summarized with percentages. Chi-square tests were performed to compare differences in categorical variables between the 2 groups.

Multivariable analyses were performed separately based on group (hemodialysis or PD). For each group, univariable analyses were first performed for each dependent variable for the outcome of interest (time from last dialysis to death, in days). Only significant variables on univariable analysis (*P* < 0.20) were included in the multivariable model. Multivariable regression Cox models were constructed, including the prespecified variables of age and sex, and significant variables (*P* < 0.20) for each group. Kaplan-Meier curves were constructed to estimate survival time until death. Missing data were excluded from analysis. There were very few missing data in the demographic variables, such as sex (0 missing values), country of birth (18 missing values), ethnicity (7 missing values), date of last dialysis (4 missing values), patient death date (0 missing values), age at death (0 missing values), primary kidney disease (14 missing values), smoking status (20 missing values), urine production at death (45 missing values), and comorbidities (all <20 missing variables). Missing data were mostly from the biochemical variables of corrected calcium (89 missing values) and phosphate (89 missing values). The values of hemoglobin, potassium, and albumin had relatively few missing data (32, 36, and 35 values missing, respectively). Only 1 patient was missing data on the cause of withdrawal.

Proportional hazards assumptions were satisfied for both the PD and hemodialysis groups. Statistical analyses were performed with IBM SPSS Statistics V26.0 (IBM, Armonk, NY). *P* values of <0.05 were regarded as significant. The reporting of this study adheres to the STROBE checklist.

## Results

### Baseline Characteristics

We included 239 patients in our study; 186 patients were on hemodialysis prior to withdrawal and 53 patients were on PD prior to withdrawal (Table 1). There was no significant difference in age of death between the 2 groups (median age 73.9 years for PD vs. 76 years for hemodialysis, *P* = 0.96) and no significant difference in proportions of men and women between the 2 groups (*P* = 0.28). The PD group had a significantly higher proportion of patients with diabetes mellitus as their primary diagnosis (62.3% vs. 38.6% for hemodialysis, *P* = 0.02), a significantly higher proportion of patients who had previously had a cancer (18.0% vs. 3.5% in the hemodialysis group, *P* = 0.01) and a significantly higher proportion of patients with urine produced at the time of withdrawal (73.6% vs. 32.8% in the hemodialysis group, *P* < 0.001). In the hemodialysis group, there was a significantly higher proportion of patients who lost >10% of their initial body weight from start of dialysis compared to their last recorded weight (56.3% vs. 27.5% in the PD group, *P* = 0.001), higher proportion of patients of Caucasian ethnicity (65.9% vs. 49.1% in the PD group, *P* = 0.04) and a significantly longer dialysis vintage (median 53.7 months vs. 33.3 months in the PD group, *P* = 0.003).

**Table 1:** Baseline demographics

Variable	Peritoneal dialysis (*n* = 53)	Hemodialysis (*n* = 186)	*P* value
Age at death, median (IQR), yr	73.9 (66.3–79.7)	76.0 (66.4–82.1)	0.96
Sex, *n* (%)			
Male	29 (54.7%)	117 (62.9%)	0.28
Female	24 (45.3%)	69 (37.1%)	
Primary diagnosis			0.02^a^
Diabetes mellitus	33 (62.3%)	71 (38.6%)	
Renovascular	8 (15.1%)	28 (15.2%)	
Glomerulonephritis	4 (7.5%)	25 (13.6%)	
PCKD	2 (3.8%)	8 (4.3%)	
Other/unknown	6 (11.3%)	52 (28.3%)	
BMI in the last 6 mo (mean ± SD, kg/m^2^)	26.2 ± 5.4	25.3 ± 8.1	0.38
Weight in the last 6 mo (mean ± SD, kg)	73.3 ± 17.3	71.4 ± 25.8	0.58
Weight loss >10% from start of dialysis to last recorded weight, *n* (%)	11 (27.5%)	85 (56.3%)	0.001^a^
SBP in the last 30 d before the last dialysis session (mean ± SD, mm Hg)	120.1 ± 34.9	124.3 ± 29.4	0.47
Region of birth			0.08
Australia and NZ	22 (43.1%)	100 (58.8%)	
Asia/South Asia	10 (19.6%)	16 (9.4%)	
Middle East	6 (11.8%)	14 (8.2%)	
Europe	5 (9.8%)	25 (14.7%)	
Pacific Islands	7 (13.7%)	10 (5.9%)	
Other	1 (2.0%)	5 (2.9%)	
Ethnicity, *n* (%)			0.04^a^
Caucasian	26 (49.1%)	118 (65.9%)	
Asian/South Asian	11 (20.8%)	22 (12.3%)	
Middle Eastern	6 (11.3%)	11 (6.1%)	
Pacific Islander	6 (11.3%)	7 (3.9%)	
Other	4 (7.5%)	21 (11.7%)	
Has ever smoked, *n* (%)	25 (50.0%)	73 (43.2%)	0.40
Chronic lung disease present/suspected, *n* (%)	10 (19.6%)	58 (32.6%)	0.07
Coronary artery disease present/suspected, *n* (%)	33 (64.7%)	114 (64.0%)	0.93
Peripheral vascular disease present/suspected, *n* (%)	34 (66.7%)	92 (51.7%)	0.06
Cerebrovascular disease present/suspected, *n* (%)	15 (29.4%)	64 (36.2%)	0.37
Diabetes mellitus present, *n* (%)	35 (68.6%)	101 (57.7%)	0.16
Has had cancer ever diagnosed, *n* (%)	9 (18.0%)	62 (3.5%)	0.01^a^
Metastatic tumor, *n* (%)	6 (11.3%)	32 (17.6%)	0.28
Myocardial infarct, *n* (%)	29 (54.7%)	103 (56.6%)	0.81
Congestive cardiac failure, *n* (%)	28 (52.8%)	94 (51.6%)	0.88
Dementia, *n* (%)	6 (11.3%)	20 (11.0%)	0.95
Peptic ulcer disease, *n* (%)	4 (7.5%)	4 (2.2%)	0.06
Hemiplegia, *n* (%)	1 (1.9%)	9 (4.9%)	0.33
Mild liver disease, *n* (%)	2 (3.8%)	8 (4.4%)	0.84
Moderate to severe liver disease, *n* (%)	0 (0%)	15 (8.2%)	0.03^a^
Charlson score (mean ± SD)	6.6 ± 2.7	7.29 ± 3.0	0.13
Urine produced at time of withdrawal, *n* (%)	39 (73.6%)	61 (32.8%)	<0.001^a^
Dialysis vintage, median (IQR), mo	33.3 (17.9–63.7)	53.7 (25.8–100.3)	0.003^a^
First dialysis modality, *n* (%)			<0.001^a^
Peritoneal dialysis	41 (78.8%)	40 (22.2%)	
Hemodialysis	11 (21.2%)	140 (77.8%)	
Previous kidney transplant (*n*, %)	0 (0%)	11 (6.0%)	0.07
Late referral to nephrologist (commenced dialysis <3 mo after referral), *n* (%)	9 (17.6%)	34 (20.1%)	0.70
Serum creatinine at start of dialysis, median (IQR), μmol/l	548.5 (461.3–638)	607 (492.5–781)	0.02^a^
Hb in the last 30 d before the last dialysis, (mean ± SD, g/l)	91.8 ± 17.4	95.6 ± 18.9	0.22
WCC in the last 30 d before the last dialysis (mean ± SD, × 10^9^/l)	11.0 ± 5.3	9.2 ± 6.2	0.05^a^
Lymphocyte count in the last 30 d before the last dialysis (median, IQR, × 10^9^/l)	0.9 (0.6–1.4)	0.9 (0.6–1.2)	0.27
Serum Na in the last 30 d before the last dialysis (mean ± SD, mmol/l)	133.5 ± 5.0	135.9 ± 4.0	0.004^a^
Serum potassium in the last 30 d before the last dialysis (mean ± SD, mmol/l)	3.8 ± 0.8	4.6 ± 0.9	<0.001^a^
Serum bicarbonate in the last 30 d before the last dialysis (mean ± SD, mmol/l)	24.9 ± 3.3	25.1 ± 3.4	0.64
Serum creatinine in the last 30 d at time of last dialysis, median (IQR), umol/l	540.5 (380.8–659.3)		N/A
Corrected calcium in the last 30 d before the last dialysis (mean ± SD, mmol/l)	2.5 ± 0.2	2.5 ± 0.2	0.21
Serum albumin in the last 30 d before the last dialysis (mean ± SD, g/l)	17.3 ± 5.6	24.0 ± 6.0	<0.001
Serum phosphate in the last 30 d before the last dialysis (mean ± SD, mmol/l)	1.55 ± 0.6	1.46 ± 0.6	0.39
CRP in the last 30 days before the last dialysis (mean ± SD, mg/l)	104.6 ± 94.7	83.3 ± 77.4	0.21

BMI, body mass index; CRP, C-reactive protein; Hb, hemoglobin; IQR, interquartile range; Na, sodium; NZ, New Zealand; PCKD, polycystic kidney disease; SBP, systolic blood pressure; WCC, white cell count.

a *P* < 0.05.

### Death Data

**Figure 1:**
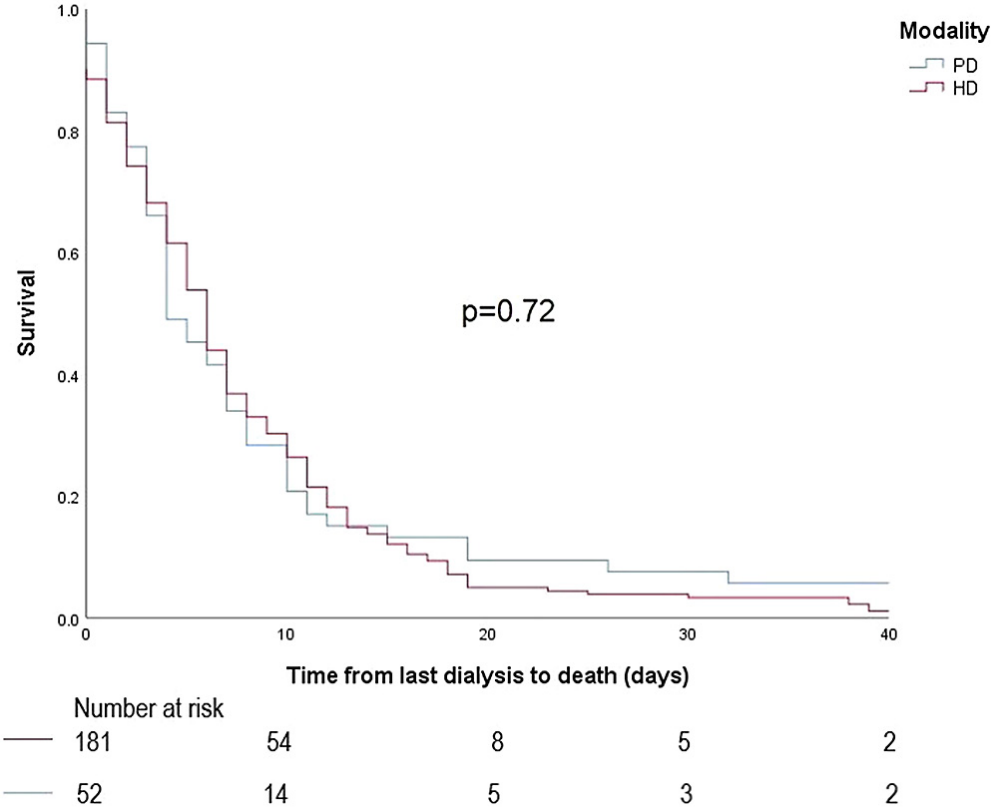
Differences in median survival after withdrawal between peritoneal dialysis and hemodialysis groups.

**Figure 2:**
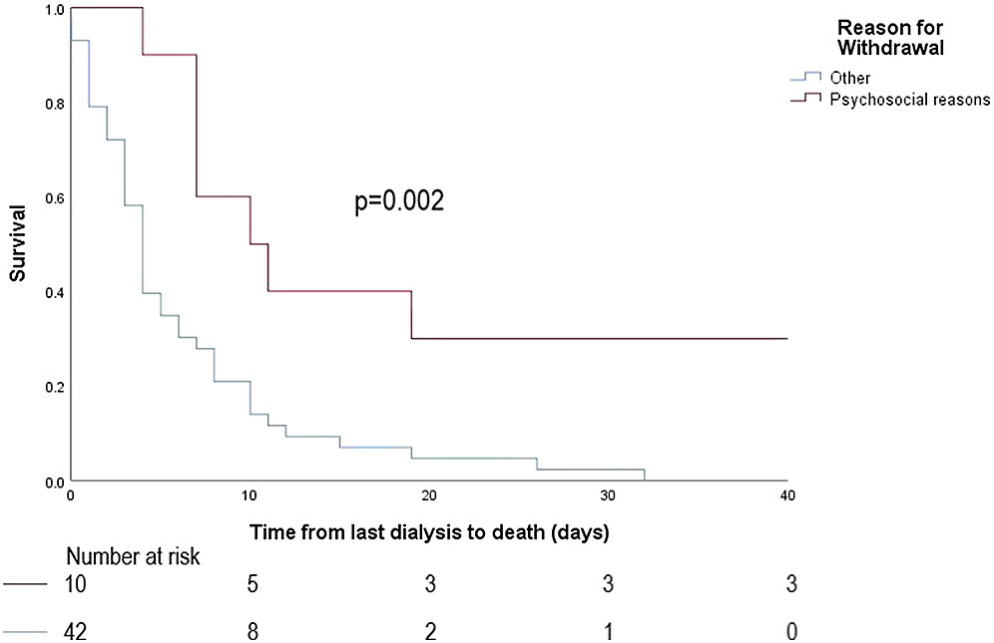
Differences in median survival for peritoneal dialysis withdrawal for psychosocial reasons versus other reasons. Median survival after withdrawal for psychosocial reason is 10.5 days versus 4 days for nonpsychosocial reasons.

**Figure 3:**
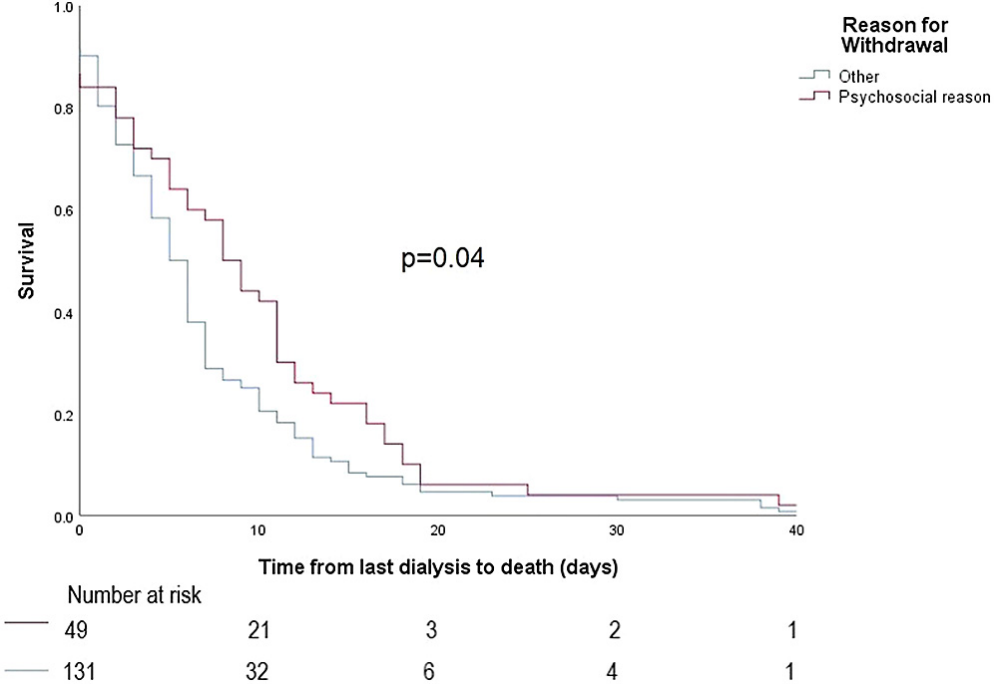
Differences in median survival for hemodialysis withdrawal for psychosocial reasons versus other reasons. Median survival after withdrawal for psychosocial reason is 10.5 days versus 8.5 days for nonpsychosocial reasons.

Death data is summarized in Table 2. Median survival time from last dialysis to death for the PD group was 4 days (IQR: 3–10 days), not significantly different from the hemodialysis group, which was 6 days (IQR: 2–11 days, *P* = 0.72; Figure 1). Withdrawal for cardiovascular reasons was the most common reason for withdrawal from dialysis in both groups. For the PD group, median survival time from last dialysis to date of death was significantly longer for patients withdrawing for psychosocial reasons compared to those withdrawing for other reasons (*P* = 0.002; Figure 2 and Table 3). In the PD group, at 7 days, 18 (34%) were still alive, and at 14 days, 7 (13%) were still alive. For the hemodialysis group, median survival time from last dialysis to date of death was also significantly longer for patients withdrawing for psychosocial reasons compared to those withdrawing for other reasons (*P* = 0.04; Figure 3and Table 3). In the hemodialysis group, at 7 days, 67 (37%) were still alive and at 14 days, 24 (13%) were still alive.

**Table 2:** Death data

Variable	Peritoneal dialysis	Hemodialysis	*P* value^a^
Time from last dialysis to death (median, IQR, ds)	4 (3–10)	6 (2–11)	0.74
Reason for withdrawing, *n* (%)			0.03^a^
Psychosocial	10 (18.9%)	50 (27.0%)	
Cardiovascular	14 (26.4%)	51 (27.6%)	
Peripheral vascular disease	8 (15.1%)	13 (7.0%)	
Cancer	2 (3.8%)	31 (16.8%)	
Neurological	7 (13.2%)	14 (7.6%)	
Other	12 (22.6%)	26 (14.1%)	
Referred to palliative care at time of death, *n* (%)	46 (93.9%)	142 (92.8%)	0.80
Year of withdrawal			0.25
2016	8 (15.1%)	28 (15.4%)	
2017	3 (5.7%)	28 (15.4%)	
2018	8 (15.1%)	16 (8.8%)	
2019	6 (11.3%)	32 (17.6%)	
2020	7 (13.2%)	28 (15.4%)	
2021	12 (22.6%)	31 (17.0%)	
2022 (until July)	9 (17.0%)	19 (10.4%)	

IQR, interquartile range.

a *P* < 0.05.

**Table 3:** Median survival from date of last dialysis until date of death

Reason for withdrawing	Peritoneal dialysis, median (IQR), d	Hemodialysis, median (IQR), d
Psychosocial	10.5 (7–107.75)	8.5 (3–13.25)
Cardiovascular	4.5 (3.75–10.25)	5 (1–7)
Peripheral vascular disease	3 (0.25–3.75)	5 (3–6.5)
Cancer	Not enough data	8.5 (5–14.25)
Neurological	7 (4–19)	5 (1–7.5)
Other	3 (2–5.75)	4 (2–10)

IQR, interquartile range.

### Multivariable Analyses

#### PD

For patients on PD, variables that were significant on univariable analysis were reason for withdrawal and C-reactive protein. Full results of the univariable analyses are shown in the Supplementary Table S1. On multivariable analysis, the only variable significantly associated with survival time from last dialysis to death was reason for withdrawing (*P* = 0.01; Table 4). Compared to the reference group (psychosocial reasons), withdrawing for cardiovascular reasons and peripheral vascular disease were significantly associated with reduced length of time from stopping dialysis until death (hazard ratio [HR]: 2.87 and 6.87, respectively; Table 4).

**Table 4:** Multivariable analyses for peritoneal dialysis group

Variable	HR (95% CI)	*P* value
Reason for withdrawing		0.01^a^
Psychosocial (reference)		
Cardiovascular	2.87 (1.12–7.33)	0.03^a^
Peripheral vascular disease	6.87 (2.33–20.26)	<0.001^a^
Cancer	3.51 (0.71–17.49)	0.13
Neurological	2.07 (0.72–5.92)	0.17
Other	5.20 (1.93–14.01)	0.001^a^

CI, confidence interval; HR, hazard ratio.

a *P* < 0.05.

### Hemodialysis

For patients on hemodialysis, variables that were significant on univariable analysis were reason for withdrawal, weight loss >10% from start of dialysis, being born in Australia or New Zealand, urine production at time of withdrawal, and serum sodium. Full results of the univariable analyses are shown in the Supplementary Table S2. On multivariable analysis, the variables significantly associated with survival time from last dialysis to death was reason for withdrawing (*P* = 0.001), urine production at time of withdrawal (*P* = 0.005), serum sodium (*P* = 0.02) and smoking status (*P* = 0.009, Table 5). Compared to the reference group (psychosocial reasons), withdrawing for a cardiovascular reason, for peripheral vascular disease or for neurological reasons were significantly associated with reduced length of time from stopping dialysis until death (HR: 2.16, 2.77 and 4.75, respectively; Table 5). Urine production and higher serum sodium were significantly associated with longer length of time from stopping dialysis until death (HR: 0.56 and 0.94 respectively; Table 5).

**Table 5:** Multivariable analyses for hemodialysis group

Variable	HR (95% CI)	*P* value
Reason for withdrawing		0.001^a^
Psychosocial (reference)		
Cardiovascular	2.16 (1.31–3.55)	0.002^a^
Peripheral vascular disease	2.77 (1.17–6.54)	0.02^a^
Cancer	1.21 (0.67–2.20)	0.53
Neurological	4.75 (2.21–10.19)	<0.001^a^
Other	1.96 (1.07–3.58)	0.03^a^
Urine produced at time of withdrawal	0.56 (0.37–0.83)	0.005^a^
Serum Na at time of withdrawal	0.94 (0.89–0.99)	0.02^a^
Ever smoked	1.66 (1.13–2.44)	0.009^a^

CI, confidence interval; HR, hazard ratio.

a *P* < 0.05.

## Discussion

In our study, the median survival time between patients withdrawing from PD and hemodialysis did not differ significantly (median survival 4 days for PD vs. 6 days for hemodialysis, *P* = 0.72). Although median survival time from last dialysis to death for our patients on hemodialysis (6 days, IQR: 2–11 days) was consistent with estimates reported in the literature (median 4 days, IQR 3–10 days,^10^ median 4 days, IQR: 2–9 days^11^), we postulated that the median survival of our PD patients would have been longer, primarily due to more retained urine output. There are no published studies estimating survival after stopping PD. However, in our cohort, the median survival of our PD patients was similar to the hemodialysis cohort. This may have been because in our study, many patients (26.4%) who withdrew from PD withdrew due to concurrent life limiting illnesses leading to acute clinical deterioration. For example, the proportion who withdrew from PD due to cardiovascular comorbidities (26%), is similar to the proportion of hemodialysis patients who withdrew due to cardiovascular reasons (27.6%). In fact, our study found that the only variable significantly associated with prognosis was the reason for withdrawal. Therefore, our data may be reflective of survival when dying from comorbid life limiting illnesses rather than uremia, rendering the advantage of urine output insignificant. It is perhaps unsurprising that in the PD population, urine output was not significantly associated with longer median survival.

Withdrawal from PD may occur later in the end-of-life course compared to withdrawal from hemodialysis. PD may be viewed as a less invasive therapy compared to hemodialysis, and able to be performed even when an individual has hemodynamic instability precluding them from continuing hemodialysis. Due to these views, it may be that clinicians and families are reluctant to cease PD until the patient has deteriorated significantly and is in the terminal phase. Thus, PD patients and their families may have little time to plan end-of-life care, particularly the logistics around hospice transfer or dying at home. Our data show that median survival after withdrawing from PD for psychosocial reasons (10.5 days, IQR: 7–107.75 days) is significantly longer than those withdrawing for medical reasons (*P* = 0.002). Cessation of PD earlier in the end-of-life phase to ease patient and family burden, when poor prognosis from medical reasons becomes apparent, may facilitate better end-of-life care planning without necessarily shortening survival.

Interestingly, only 18.9% of PD patients in our study withdrew for psychosocial reasons. This contrasts with withdrawal data from Australia and New Zealand in 2020,^15^ in which 40.4% of patients withdrew from PD for psychosocial reasons and only 25.2% withdrew for cardiovascular reasons. This may be explained by demographic or cultural differences; however, we were not able to further explore this in our data. Further work would need to be done to determine the effects of cultural background on withdrawal decisions.

For patients withdrawing from hemodialysis, variables significantly associated with prognosis were reason for withdrawal, urine production at time of withdrawal, serum sodium at time of withdrawal, and smoking status (current, past, or nonsmoker). Previous studies have identified male sex, lower functional status, the presence of peripheral edema, presence of pleural effusions, hypoxemia, and poor oral nutrition as factors significantly associated with shorter prognosis after withdrawal.^9^^,^^11^ In our study, higher serum sodium was associated with a longer survival time from stopping dialysis until death (HR: 0.94 for each 1 mmol/l increase in serum sodium, 95% confidence interval [0.89–0.99]). Higher serum sodium may be an indicator of fluid status and less fluid overload, because the studies above have described indicators of fluid overload such as edema and pleural effusions correlating with shorter survival. The variables that we have identified in our study have not been previously described in the literature and are practical clinical indicators that can be easily assessed at the time of dialysis withdrawal to lend valuable prognostication data to patients and their families.

In both groups, withdrawing due to medical reasons such as cardiovascular disease and peripheral vascular disease (HR: 2.87 and HR: 6.87, respectively for the PD group; and HR: 2.16 and HR: 2.77 for the hemodialysis group) were associated with reduced survival, compared with psychosocial withdrawals. Reduced median survival from these conditions may indicate death from comorbid life threatening medical conditions rather than uremia. Notably, palliative care referral was >90% in both PD and hemodialysis patients withdrawing from treatment, much higher than noted in previous studies (34%).^16^ This may suggest that with palliative care supports available, patients who face the prospect of imminent death from medical conditions are willing to cease active treatment under the guidance of their clinicians. Furthermore, these data aid counseling and individualized planning for end-of-life care. Clinicians may utilize this information to better counsel patients regarding prognostic expectations and median survival after withdrawal, which may differ depending on certain clinical factors. Given that those withdrawing for cardiovascular or peripheral vascular disease reasons can be expected to have a shorter median survival, clinicians caring for such patients may wish to initiate end-of-life planning discussions sooner in the clinical course because this would have implications on a patient and family’s end-of-life care plans.

Strengths of our study include a comprehensive data set with few missing values and the inclusion of variables relevant to a renal population, which are normally difficult to collect in an end-of-life setting such as urine output. This study is the only study to our knowledge to specifically examine prognostication in a population withdrawing from PD. Previous studies examining a similar question only included hemodialysis patients. Limitations of our study include a small cohort size overall, particularly in the PD group, which limits statistical power to detect prognostic factors with a potential risk of overfitting the PD model. There may also be reporting bias, because the data collected is staff dependent, particularly on what is deemed “psychosocial” withdrawal. Our patient cohort differed in terms of some baseline demographics compared to the overall Australian and New Zealand demographic data, including a lower prevalence of Caucasian patients, and a higher prevalence of diabetes mellitus type 2, which may have played a role in why the main cause of withdrawal was withdrawal from cardiovascular comorbidities. The study is also limited by its retrospective nature because we were unable to accurately collect variables such as volume status or functional status that likely impact upon prognostication. Future research should endeavor to determine if these significant prognostic factors remain significant in different population groups.

### Conclusion

As withdrawal of dialysis becomes more common, clinicians require information pertaining to prognostication after withdrawal for counseling both patients and their families. Our study describes the median survivals after discontinuation for both PD and hemodialysis populations, and differences in survival depending on the reason for withdrawal. Future research could further define survival, symptom trajectories, and palliative care utilization after dialysis discontinuation to further aid shared decision making discussions and end-of-life care planning.

## Disclosure

All the authors declared no competing interests.

## Data Availability Statement

The datasets used and analyzed during the current study are available from the corresponding author on reasonable request.

## Author Contributions

SS contributed research idea and study design, data acquisition, data analysis/interpretation, and manuscript write-up. KCLL did manuscript revision. Each author contributed important intellectual content during manuscript drafting or revision and agrees to be personally accountable for the individual’s own contributions and to ensure that questions pertaining to the accuracy or integrity of any portion of the work, even one in which the author was not directly involved, are appropriately investigated and resolved, including with documentation in the literature if appropriate.

## Supplementary Material

Supplementary File (PDF) Table S1. Univariable analyses for peritoneal dialysis group. Table S2. Univariable analyses for hemodialysis group. **STROBE Statement.**

## Footnotes

Supplementary File (PDF)
**Table S1.** Univariable analyses for peritoneal dialysis group. **Table S2.** Univariable analyses for hemodialysis group.
**STROBE Statement.**

## References

[1] Khou V., De La Mata N.L., Morton R.L., Kelly P.J., Webster A.C. (2021). Cause of death for people with end-stage kidney disease withdrawing from treatment in Australia and New Zealand. Nephrol Dial Transpl.

[2] van Oevelen M., Abrahams A.C., Bos W.J.W. (2021). Dialysis withdrawal in the Netherlands between 2000 and 2019: time trends, risk factors and centre variation. Nephrol Dial Transpl.

[3] Findlay M.D., Donaldson K., Doyle A. (2016). Factors influencing withdrawal from dialysis: a national registry study. Nephrol Dial Transpl.

[4] Birmelé B., François M., Pengloan J. (2004). Death after withdrawal from dialysis: the most common cause of death in a French dialysis population. Nephrol Dial Transpl.

[5] Chan H.W., Clayton P.A., McDonald S.P., Agar J.W., Jose M.D. (2012). Risk factors for dialysis withdrawal: an analysis of the Australia and New Zealand Dialysis and Transplant (ANZDATA) Registry, 1999-2008. Clin J Am Soc Nephrol.

[6] Chen J.H.C., Brown M.A., Jose M. (2022). Temporal changes and risk factors for death from early withdrawal within 12 months of dialysis initiation-a cohort study. Nephrol Dial Transpl.

[7] Wachterman M.W., Pilver C., Smith D., Ersek M., Lipsitz S.R., Keating N.L. (2016). Quality of end-of-life care provided to patients with different serious illnesses. JAMA Int Med.

[8] Chu C., White N., Stone P. (2019). Prognostication in palliative care. Clin Med.

[9] Davison S.N., Rosielle D.A. Palliative Care Network of Wisconsin. Clinical care following withdrawal of dialysis. https://www.mypcnow.org/fast-fact/clinical-care-following-withdrawal-of-dialysis/.

[10] Yamaguchi K., Kitamura M., Takazono T. (2022). Parameters affecting prognosis after haemodialysis withdrawal: experience from a single center. Clin Exp Nephrol.

[11] O’Connor N.R., Dougherty M., Harris P.S., Casarett D.J. (2013). Survival after dialysis discontinuation and hospice enrollment for ESRD. Clin J Am Soc Nephrol.

[12] Prompantakorn P., Angkurawaranon C., Pinyopornpanish K. (2021). Palliative performance scale and survival in patients with cancer and non-cancer diagnoses needing a palliative care consultation: a retrospective cohort study. BMC Palliative Care.

[13] Couchoud C., Labeeuw M., Moranne O. (2009). A clinical score to predict 6-month prognosis in elderly patients starting dialysis for end-stage renal disease. Nephrol Dial Transpl.

[14] Ferreira E.S., Moreira T.R., da Silva R.G. (2020). Survival and analysis of predictors of mortality in patients undergoing replacement renal therapy: a 20-year cohort. BMC Nephrol.

[15] Australian and New Zealand Dialysis and Transplant Registry. Chapter 3: mortality in kidney failure with replacement therapy. ANZDATA Registry. http://ANZDATA_AR-2022-23_Chapter-3_F5.pdf.

[16] Chen J.C., Thorsteinsdottir B., Vaughan L.E. (2018). End of life, withdrawal, and palliative care utilization among patients receiving maintenance haemodialysis therapy. Clin J Am Soc Nephrol.

